# Clinical Reports on Continued Eever

**Published:** 1852-11

**Authors:** 


### REVIEWS.

---

Art, IV.—*Clinical Reports on continued fever, based on an analysis of one hundred and sixty-four cases; with remarks on the management of continued fever; the identity of typhus and typhoid fever-relapsing fever, diagnosis, etc. To which is added a memoir on the transportation and diffusion, by contagion, of typhoid fever, as exemplified in the occurrence of the disease at North Boston, Erie county, N. Y.* By Austin Flint, M. D., Professor of the Principles and Practice of Medicine, and of Clinical Medicine, in the University of Buffalo, and Editor of the Buffalo Medical Journal. Buffalo: George H. Derby & Co., 1852.

Dr. Rush was wont to say that a man might as well attempt to portray a summer cloud in a painting, as to attempt to describe a fever. The elements of the disease are undergoing perpetual changes, and Dr. Rush looked upon any attempt at seizing and fixing the fleeting movements as an attempt at impossibilities. Those who have devoted their lives to the perusal of treatises on fever, may easily conclude that Claude, of Lorraine, was more successful in fixing the features of summer clouds on canvass, than writers on fever have been in portraying its changing forms. Doctors differ widely in their views, and their differences have given point to a sarcasm. But these differences exist in the varied phases of the human mind, and are not confined to the medical profession. Two artists may view the same landscape, and in their attempts to transfer their impressions to canvass, may differ so widely that it would be difficult for any one to detect a resemblance. Yet, each may have been honest in his attempts, and faithful in executing his conceptions. Fixed as the features of a landscape may seem to be, a thousand circumstances may change the appearances that strike the eye. That which was dressed in peculiar loveliness, while the first beams of the morning sun bathed themselves in the glistening dew, or lighted up the glossy green of the grass, or enlivened the beauty of the flowers, would, in the roseate blush of twilight, look like a different scene. While the bloom of the Drummond Phlox is in repose, it presents a uniform mass of red; but if the passing zephyr shall curl its beauties, the prismatic colors might envy the varied hues that dance upon the retina of the eye. It is evident, then, that men may differ without mistaking matters. If we wish to do their differences justice, and drink in truth ourselves, we must know how each conception was formed; we must know from what point each viewed the landscape, and the circumstances that governed the view.

If in such a comparatively permanent fixture as a landscape, hese considerations should weigh, how greatly must they be extended when they come into the range of fever, where all is change; where multitudes of circumstances are fixed in nothing but their power to cause variableness. The time may come when all this variety may classify itself, and men may be able to arrange the movements of changeableness into positive groups, as the geologist does his formations. Confusion once reigned supreme in geological science, where now the human intellect has planted beacons that give perpetual light; and if the proper methods of observing and analyzing fever are kept up, we doubt not the path of progress will be well worked.

In the treatise before us, Professor Austin Flint has presented a notable example of patient observation and industry, of clear and comprehensive views, and of rigid analysis. In undertaking to exhibit his labors, we wish it to be distinctly understood that it must not be taken for granted, that merely because we do not at any point controvert the views or inferences of the author, we necessarily agree with him. Our main object is to point the attention of the reader to the views of Professor Flint, and to show what they are, and occasionally to refer to the foundations on which they rest.

The Clinical Reports originated in the fact that at the annual meeting of the New York State Medical Society, in 1850, the scientific subject which elicited most discussion was the identity or non identity of the forms of fever now commonly known as *Typhus* and *Typhoid;* and a committee was appointed on which the author was placed as chairman, to collect facts relating to Continued Fever, and report at the next annual meeting. The assignment of this duty led the author to examine the cases occuring under his own observation, the histories of which he had recorded ; and finding the number considerable, he concluded to subject them to numerical analysis,—and these, for good and sufficient reasons, were published in successive numbers of the Buffalo Medical Journal, and afterwards presented in that shape as a report to the society. In the autumn of 1850, and winter of 1850–'51, another series of cases passed under observation at the *Buffalo Hospital of the Sisters of Charity*, where most of the cases forming the basis of the report just mentioned had been observed. In order to compare the results of the analysis of this second collection with those of the first, the author undertook the labor in the summer of 1851, and published the results in the Medical Journal in which the first series appeared. This second collection consisted of forty-eight cases. As a supplement to these reports, some remarks were contributed on the "Symptoms of Typhoid and Typhus Fever ; the identity of these two types ; the diagnosis, etc." A paper on the "management of continued fever," was also added. In the autumn of 1851, and the winter of 1851–'52, another series of cases more numerous than before, passed under the observation of the author. This series constituted the "Third Clinical Report on Continued Fever, based on an analysis of sixty four cases." Appendices were added to the third report. The first is on the morbid condition after death, (exclusive of intestinal lesions,) distinctive of Typhus and Typhoid fever. The facts in this paper are derived from Dr. Jenner's publications in the Edinburg Monthly Journal of Medical Science, in a series of articles commencing with the No. for April 1849, and ending with the No. for April 1850. This series presented an analysis of sixty-six cases of Continued Fever, embracing the two types, Typhoid and Typhus, "the object being to settle the question of the identity or non-identity of the two types by a comparison of the phenomena presented during life, and disclosed with the scalpel after death. The second appendix relates to *Relapsing* fever, and presents the results of an analysis of fifteen cases.

The closing paper of the work is devoted to a "Memoir on the Transportation and Diffusion by Contagion, of Typhoid fever, as exemplified in its occurrence at North Boston, Erie county, New York."

We have thought it due to the author, and to the important subject to give a minute detail of the sources of information that form the basis of the work. No one can doubt that the course pursued by Professor Flint is the correct one for shedding light upon a subject which has many points of obscurity in it, notwithstanding the vast number of tomes that have been written for the special purpose of clearing up all obscurity. The reader of the present work will find himself in the hands of a remarkably intelligent observer, whose teachings deserve and must command respect. The work is in fact a series of Clinical lectures in which an able observer discourses clearly and intelligently upon the phenomena of disease, as they spring to view in the progress of the disease, and they are filled with a fund of instruction that cannot well be taught even at the bed-side.

We do not purpose to enter into an analysis of the statistical details made by Professor Flint. The mere statistics could not be made very profitable to the reader, without an ample reference to the details on which the statistics are founded. Dr. Flint has so perfectly interwoven the various parts of his subjects, that it would be difficult to select any particular part as a sample. But we hope that those who desire to understand all that can be known about the types of continued fever, will not neglect the important contribution made to the subject in this work. It is only by the study of every possible phase of this disease, under the observation of honest, intelligent, and reliable observers, that any one can hope to recognise the ever-varying forms it assumes. These Clinical Reports are very full, and will well reward the diligent student. In order that the reader may see something of the completeness of the plan of observation, we present the elements of the various sections;

Section 1. Age; occupation; civil condition; nativity; habits; season; constitution and previous health of the patient; period of residence in this country, and in this city; duration of the disease before coming under observation.

Sec. 2. The access; Its duration and symptoms; circumstances supposed to have been concerned in the production of the disease.

Sec. 3. Symptoms referable to the general aspect; expression of countenance; decubitus.

Sec. 4. Symptoms referable to the nervous system; mind; sleep; coma; senses and sensibility; muscular contractions, etc.

Sec. 5. Symptoms referable to the digestive system; appetite; thirst; tongue; sordes; parottitis, nausea and vomiting; alvine djeetions; tympanitis; tenderness of abdomen; gurgling.

Sec. 6. Cutaneous eruptions.

Sec. 7. Symptoms referable to the respiratory apparatus; cough; expectoration; pain in the chest; pneumonitis; aberration of respiratory movements; epistaxis; singultus.

Sec. 8. Symptoms referable to the circulation; pulse; capillary congestion.

Sec. 9. Symptoms (exclusive of eruptions) referable to the skin.

Sec. 10. Symptoms referable to the genito-urinary system.

Sec. 11. Duration of the disease; circumstances attending convalescence; sequelæ; mode of dying; fatality.

Sec. 12. Examinations after death.

Sec. 13. Treatment.

Sec. 14. Cases of doubtful type.

The reader will at once perceive, even from these indicæ, how very comprehensive a scope Dr. Flint has taken in making these Clinical Reports. The objects of investigation, embraced in these subjects, belong to each of the three Reports.

The reader may be desirous of knowing the course of treatment pursued by Professor Flint, and to that we direct attention. The means used are distributed under their appropriate heads.

In *two* cases, both of the *Typhus* type, detailed in the third Clinical Reports, no medicinal remedies were employed. Sanitary measures alone were ordered, and the cases terminated favorably.

*Laxatives.* These were prescribed in several cases where there had been no dejection for several days. No active cathartic was administered in any case. Half ounce doses of castor oil by the mouth, or in simple enemas, were the laxative measures. The oil was used in ten cases. Nine of these were of the typhus type. In no case, but one, was the oil given more than once during the course of the disease. In nearly every case it was given early in the disease. A single enema was prescribed in *three* cases, all of the *Typhus* type. In one case where castor oil was given, and in one in which an enema was resorted to, the dejections which followed were so frequent that restraining means were necessary.

*Anodynes.* These were employed in a few cases to restrain diarrhœa, and the undue operation of laxative remedies. The tinct. opii. was used, taken either by the mouth or by enema. It was resorted to in five cases of *Typhus*, and eight cases of Typhoid. The use was very limited. The author had been accustomed to the prescription of anodynes to relieve mental aberration, and promote sleep. Even where vigilance or delirium was not a prominent symptom, Dr. Flint had supposed an anodyne influence salutary. But in the class of cases embraced in the third clinical report, anodynes were resorted to exclusively for restraining undue activity of the bowels, and the sense of their necessity for any other purpose was lessened by the observation of cases in which they were omitted.

*Astringents.* Tannic acid was given in only *one* case of *Typhoid*, to restrain diarrhœa.

*Sedatives.* The articles employed under this head were *tartarized Antimony* and *Potassa*, and *Camphor*. The former was used in *three* cases of *Typhus*, and in one case of *Typhoid*. In the first class it was given in doses of 1–16th of a gr., to relieve delirium. In the *Typhoid* case it was given for pneumonitis. In one case of *Typhus*, the antimony constituted the sole medicinal treatment. *Camphor* was given in three cases of *Typhus* for taxic symptoms, and in no case of *Typhoid*.

*Ammonia.* The carb. of Ammonia was given whenever the circulatory forces were failing. It was used in all the cases ending fatally by Asthenia. It entered into the treatment of six cases of *Typhus*, and four cases of *Typhoid.*

*Quinia.* This was prescribed in doses of *three* grains, three times daily, and continued several days in *four* cases of Typhus. In each of these cases it was given from the commencement of the career. It was given in one of Typhoid.

*Huxam's Tinctnre* was prescribed in *one* case.

*Vesication.* Blisters to the nape of the neck were resorted to whenever spasmodic inspiration was observed, or a tendency to coma. They entered into the treatment of all cases that ended fatally by apnœa. The only exception of their use for other symptoms than those named, was their application behind the ears for external otitis. They were applied to the neck in *nine* cases of *Typhus*, and in four cases of *Typhoid.*

*Sinapisms and stimulating liniments.* Occasionally employed where pneumonitis was the complication, or where coma existed or was threatened.

*Diffusible Stimulus.* Brandy was almost the sole agent employed as a diffusible stimulus. It entered into the management of most of the cases of both types. It was omitted in but five cases of *Typhus*, and two of *Typhoid.* In several of the Typhus cases (in all nine) brandy constituted the sole remedial agent employed. The quantity given was generally half an ounce; in some cases an ounce. The intervals of its administration varied from half an hour to four hours. In a large proportion of cases, the remedy was commenced early in the disease, and continued to its close. Prostration, coolness of the surface, feebleness, and, more especially, notable frequency of the pulse were regarded as indicating its free administration. But it was often given to forestall, by sustaining the vital forces, the occurrence of these symptoms.

Nutriment was systematically administered, such as essence of beef and milk porridge. The articles of nourishment were given sometimes against the inclinations of the patients. Cleanliness, ablutions and ventilation, were resorted to.

The only attempts at cutting the disease short, were made with the *wet sheet*, and it was used in two cases of *Typhoid* fever without any useful result.

These various elements of treatment exemplify, therapeutically, the treatment of *Typhus* and *Typhoid* fever mainly by stimulants and nourishment. The rate of mortality was not large.

We present the following synopsis, inDr. Jenner's language, in order that our readers may see what that eminent practitioner has to say on the important subject of morbid appearances distinctive of Typhus and Typhoid fever, based on an analysis of sixty-six cases:

" *Cadaveric rigidity*, ceased much more quickly in subjects dead from typhus than from typhoid fever."

" *Discoloration of the walls of the abdomen and of the skin covering the larger veins*, was much more frequently present in those dead from typhus than typhoid fever."

" *Emaciation* had made greater progress in the typhoid than in the typhus subjects."

" *Spots.* The spots observed during the progress of the cases of typhus fever continued after death; no trace of the spots visible during life could be detected after death from typhoid fever."

" *Head.* After typhoid fever, the pia mater and arachnoid separated from the convolutions with abnormal facility in one only of nine cases examined with reference to this point. The vessels of the pia mater were abnormally filled with blood in one-third of the cases, but intensely congested in one only of the fifteen cases; the cerebral substance was congested in one-seventh of the cases. After typhus fever, the pia mater and arachnoid separated with abnormal facility in nine of eleven cases of which notes on the point were made. The vessels of the pia mater were congested in nearly half, and intensely congested in one-fifth of the whole of the cases; while the cerebral substance itself was abnormally congested in half."

"*Hemorrhage into the cavity of the arachnoid,* which was not found in a single case of typhoid fever, had occurred before death in one-eighth of the cases of typhus fever." "The amount of serosity found within the cranial cavity was decidedly greater after typhus than typhoid fever."

"*Pharynx.* After typhoid fever, this organ was found ulcerated in one-third of the cases. After typhus fever, ulceration of the pharynx was not detected in a single case."

"*Larynx.* Ulceration of the larynx was found in one of fifteen subjects dead from typhoid fever; in one of twenty-six from typhus fever."

"*Œsophagus.* After typhoid fever, ulcerated in one of fifteen cases in which it was examined. After typhus fever, the œsophagus was free from ulceration in all the twenty-four cases in which it was examined."

"The epithelium separated from the œsophagus spontaneously at an earlier period after death from the latter than the former disease."

"*Stomach.* In none of the fifteen cases examined after death from typhoid fever was the mucous membrane of the stomach softened throughout the whole extent; in no case did softening of the cardial extremity approach perforation. In four of thirty-seven cases of typhus fever the whole mucous membrane of the stomach was softened; and in four others there was such extreme softening of the whole of the coats of the great *cul de sac,* that they were perforated by the slightest violence.

"*Small Intestines and Mesenteric Glands.* The presence or absence of lesion of these organs was the ground on which the cases of typhoid and typhus fever here analyzed were divided from each other—consequently they were invariably diseased in the one, and sound in the other."

"*Large Intestines.* After death from typhoid fever the mucous membrane of the large intestines was found ulcerated in rather more than a third of twenty cases In no instance after death from typhus fever.

" *Peritoneum.* As peritonitis was in typhoid fever sec ondary to, and dependent on the entero mesenteric disease, it may here be excluded from consideration."

" *Spleen.* This organ was enlarged in all the cases of typhoid fever—softened in one-third of the cases only. Before the age of fifty, it was as large after typhus as typhoid fever; after that age, it was decidedly smaller in the former than in the latter affection. After the age of fifty, it was as soft in typhus as in typhoid fever; before that age it was frequently softened."

" *Gall Bladder.* There was ulceration of the lining membrane of the gall bladder in one of fourteen cases of typhoid fever; in none of thirty-one cases of typhus fever. In the latter disease the bile was much thicker, and of a darker green color than in the former."

" *Liver, Pacreas, Kidneys.* These organs were more flabby in the cases of typhus than in those of typhoid fever."

" *Ordinary Bladder.* This viscus was ulcerated in one of the cases of typhoid fever—in none of the cases of typhus fever."

" *Pericardium.* This cavity contained a small amount of yellowish transparent serosity in all the cases of typhoid fever examined. The contained serosity was red, from transudation of a solution of hæmatosin, in five of thirty-one cases of typhus fever, in which the pericardium was examined before the termination of the fever."*

" *Heart.* The muscular tissue of this organ was much more frequently and decidedly flabby, and its lining membrane was much more frequently and deeply stained of a dark red color, in the cases of typhus fever, than in those of typhoid fever."

" *Lungs.* Granular and non-granular lobular consolidation were very frequent in the subjects dead from typhoid fever—rare in those dead from typhus fever. The reverse was the fact with reference to congestion of the most depending part of the lung."

" *Pleura.* Recent lymph or turbid serosity was found in six of fifteen cases of typhoid fever—i. e., between half and one-third, or in the proportion of forty per cent.

* There is evidently a mistake in the last sentence.

The same lesions not much less in amount were found in two only of thirty-six cases of typhus fever—i. e., one-sixteenth, or in the proportion of fifty-five per cent."

The foregoing is copied from the Monthly Journal of Medical Science for April, 1850.

Dr. J. adds, after the foregoing parallel of pathological appearances, as follows: "The particulars here briefly recapitulated, and still more those fully detailed in the foregoing papers, appear to me to prove indisputably that the symptoms, course, duration, anatomico-pathological lesions, and the tendency to cadaveric changes, are different in typhoid fever from what they are in typhus fever."

On the subject of relapsing fever we should like to present a full analysis of the views of Professor Flint, but we must content ourselves with snch a notice of this form of fever as will enable our readers to observe and detect it. We have seen several cases of this apparent mongrel variety of fever, and its phenomena are at times very embarrassing. The following is the synopsis made by Professor Flint:

Evidences of a form of fever supposed to be identical with *Relapsing fever*, have heretofore received different names, such as the "short fever;" the "five day fever;" the "seven day fever;" "bilious remittent fever;" "remittent icteric fever;" "mild yellow fever," etc.

The disease is stated to affect all ages, and both sexes in about an equal ratio.

The *access* does not present any very distinctive features. The attack is oftener abrupt than in typhoid fever, and the muscular and articular pains are apt to be severe.

Delirium, and other head symptoms are represented to be oftener absent, and, when present, less in degree than in the other forms of continued fever.

The absence of the abdominal symptoms which are so generally present, in a greater or less degree, in *typhoid* fever, is an important point distinguishing *relapsing fever.* Diarrhœa, with notable meteorism, and tenderness in the iliac regions, do not belong to the latter. It is, however, characterized by certain symptoms pertaining to the digestive system rarely found in *typhoid*, viz: nausea and vomiting, which are frequently prominent symptoms, and tenderness over the epigastrium. The matter vomited is stated to be "bright grass green," and sometimes like coffee grounds, approximating to the black vomit of yellow fever.

The eruptions characteristic of *typhus* and *typhoid*, are not found in *relapsing* fever. Sudamina and occasionally petechiæ have been observed. An eruption consisting of "small spots, round, purple, unaltered by pressure," has been described by some observers as an element of the affection. These spots are stated to be extremely like flea bites, and it does not appear to be fully ascertained that they were not, in fact, flea bites. The chest symptoms, i. e.: cough and bronchial rales, so generally present in *typhoid* fever, are said to be less commonly present in this disease. *Epistaxis*, so frequently observed in *typhoid* fever, sometimes occurs. The pulse, seldom falling below 100, in more than half the cases is 120, and in a considerable number the frequency is still greater.

Profuse sweating is stated to occur pretty uniformly, preceding the apparent convalecence and also when the relapse of fever is about to terminate. Another distinguishing feature referable to the skin, is more or less yellowness, occuring on the fourth or fifth day. In severe cases the jaundice is often a prominent symptom. The most distinctive event, however, is that indicated by the title of the disease, to wit: the occurrence of relapses. The first febrile career continues for a period rarely less than *four*, nor more than *ten* days, and then ends, generally after profuse sweating, which is considered to be critical, leaving the patient free from febrile movement, and apparently convalescent. After an interval varying, in duration from *five* to *eight* days, another attack occurs, generally abruptly, and frequently preceded by a chill.—The recurring febrile movement is equally or more severe than the first. It continues from four to five days, and then terminates usually after profuse sweating. Usually after a single relapse, the patient becomes permanently convalescent. A second, third, and even a greater number of relapses, however, have been observed to occur.

The disease is seldom fatal. Patients almost uniformly recover, unless some complication takes place, such as pleurisy, pneumonitis or dysentery.

The most distinctive characters derived from autopsical examinations, are the absence of the intestinal lesions belonging to *typhoid* fever. The peyerian glands are unaffected. The spleen is generally enlarged and softened.

It may be communicated by contagion, and Dr. Jenner, in the article already referred to, has collected observations tending to show that relapsing fever cannot be derived from patients laboring under the other forms of continued fever, but that it alone produces the special miasm for its propagation. How far the facts contributed by him go to establish this important point, it does not fall within my present purpose to inquire.

This form of fever does not secure immunity from the other forms; nor from subsequent attacks of the same form.

These are, briefly, the more prominent of the traits distinctive of *relapsing* fever which are mentioned by the two writers to whom I am indebted for the foregoing summary. They appear to have been deduced from an examination of reports of epidemics, supposed to be of this character, by various authors. If they are sufficient to render probable the position that the disease to which they relate is a peculiar form of fever, distinct from the *typhus* and *typhoid* forms, it is certainly desirable that collections of cases should be subjected to careful numerical analysis. The question can only be definitely settled by the results of such analysis.

So far as I know, we have no account of a form of fever prevailing in this country, analogous to the relapsing fever as just described. Were the cases characterized by relapse, among those observed by me during the winter of 1850–51, cases of *relapsing* fever? With a view to arrive at the answer to this question, and, if the conclusion be in the affirmative, to furnish a small contribution toward the natural history of that form of fever, I will now proceed to analyze the cases referred to.

We must here close our analysis of this valuable contribution to the subject of Fever, and pass to the consideration of Professor Flint's paper on "The Transportation and Diffusion by Contagion of Typhoid Fever." There is nothing about Typhoid fever that has called forth a greater variety of opinions than the means of its transportation and diffusion. How far the epidemic character of Typhoid fever may sustain the doctrine of contagion, we are not prepared to say. We must frankly say that we have never seen any thing, under our own observation, that would justify a belief in contagion. We think the disease is somewhat rare in Louisville, because we have seen but little of it here in the course of many years. Very recently we saw four cases in one family. In this instance we know of nothing that would lead any one to suspect contagion. There were no cases from whence these subjects might have caught the disease, and numerous children visited their sick friends, but no one of them received any infection. There were young members of the family exposed to whatever there may be of infection in the disease, but they did not suffer from the exposure. These are facts that we observed, but we do not urge them as militating against the doctrine of contagion; we mention them only as facts observed. They are too limited either to establish or destroy a doctrine.

It is evident, however, that the belief in the contagiousness of Typhoid fever is growing, and we are assured that there are observers, who commenced making memoranda, as anti-contagionists, who are surprised to find facts accumulating among their records, that strongly point to contagion. While we remain unconvinced, we acknowledge that there are appearances in favor of the contagiousness of the disease, that cannot well be accounted for or explained if we deny the doctrine of contagion. The paper of Professor Flint, of which we are about to make a synopsis, has already converted former skeptics, and fixed the faith of some who were wavering.

It is due to the subject that the facts drawn from the fever of North Boston, Erie county, N. Y., be fairly presented.

North Boston is about eighteen miles from Buffalo, and twelve miles from the shore of Lake Erie. It was a salubrious spot. No malarious source was known in its neighborhood. Neither Intermittent fever, nor any of its kindred types, had prevailed for several years. Typhoid fever had never appeared in any part of Erie county. Remittent fevers had been the usual types, but these have diminished, and Typhoid fever has proportionally increased.

In 1843, the year of the fever, there were but nine families in North Boston, all living within an area of an hundred rods in diameter; but the few houses in which the disease occurred were closely grouped together, the house farthest removed from the tavern being only ten rods distant. Forty-three persons made up the entire community.

In September, 1843, a young man from Massachusetts being on a journey westward, took lodgings at Fuller's tavern. He had been ill for several days, and stopped at North Boston because he was unable to proceed any farther. He remained at the tavern, and died on the 19th of October. The symptoms establish the fact that it was a case of *typhoid* fever. In the portion of Massachusetts from which the stranger came that is the prevailing form of fever.

Between October 19th and December 7th, twenty-eight of the forty-three inhabitants of North Boston were attacked with fever, and ten of the cases proved fatal. The first case was in a son, aged sixteen, of the inn-keeper. He was attacked twenty-three days after the arrival of the stranger. About the same time a daughter of the inn-keeper was taken ill with the fever; then another daughter, about twelve days later, and another aged three years, about the same time. Another son of the inn-keeper was attacked on the 9th October. Two othe cases occurred in this family. Seven cases, exclusive of the stranger, occurred in this family.

A son of the inn-keeper, a married son, living about four rods from the tavern, who visited his father's in order to assist in nursing, was attacked on the 15th of October.

About eight rods from the tavern a family of the name of Penhallow was attacked. In the family of Ingraham, residing about three rods from the tavern, were seven cases and three deaths. A family named Hallick resided about ten rods from Fuller's, and seven cases occurred in this family, of which five were fatal. A family named Dobbs, about three rods from the tavern, had four cases, all of whom recovered. Another case occurred in a family living within twenty feet of the tavern.

Only three families in the settlement escaped. One of these lived in a part of the house occupied by the inn-keeper's married son. This was the only family in the restricted area of the disease that escaped. Another family, named Sprague, lived about forty rods from the tavern. Bailey's family also lived about forty rods from the tavern.

In the families that were attacked there were individuals who escaped. Their names and ages are as follows: Fuller, the inn-keeper, about 50; Ingraham, about 50; Dobbs, about 45; Penhallow, about 40, and Hallick, about 40. Fuller was in comfortable circumstances. The other families were poor, almost to destitution. The families who escaped were in good circumstances.

In the mysterious circumstances attendant upon this crushing and new calamity, the imagination rioted.—Wiseacres, as usual, settled, to their own satisfaction, a problem that puzzled philosophers. Untrammelled by those absurd rules that logicians call laws, these popinjays jumped to conclusions without undergoing the tediousness of a journey of ratiocination. Their dictum was sufficient, and all were fools who did not leap over such things as facts, and seize their doctrine without the trouble of reasoning. These jumping sages found the cause of this disease of New Boston in a poisoned well, just as their similitudes in the dark pestilences of Europe always found the cause of epidemics in water-fountains. They probably had heard that truth lies in the bottom of a well, and for either that reason, or probably for not as good a reason, located the origin of pestilences in wells and springs.

In New Boston the family of Fuller, where the disease commenced, and the family of Stearns, who escaped an attack, were engaged in a bitter feud, and as the disease was ascribed to poison, administered by Stearns, all the families of the settlement cut the acquaintance of his family. A well, near the tavern, was used by all the families except that of Stearns and one other family. It was charged that Stearns had poisoned the well, and futile efforts were made to empty it with pumps. Up to a short period before the outbreak of the disease, the family of Steanes had been supplied with water from Fuller's well, but, owing to the feud referred to, the family of the former had been cut off from the well, and had been forced to make a well for themselves. Another of the three families that escaped did not use the water of Fuller's well. By the others it was used daily. Upon these coincidences the assumption was formed that Stearns had poisoned the well. Some of the water was taken to Buffalo and analysed. The water was found to be remarkably pure. The excitement against Stearns was of course very great, and he instituted a prosecution for slander, which was settled by the party prosecuted by the payment of one hundred dollars.

The mysterious character of the disease, its unusual phenomena, and the poverty of the sufferers, called for medical aid from Buffalo, and Dr. Flint visited North Boston. He satisfied himself by extended observations, and patient examination, that the disease was Typhoid fever. Autopsies revealed the characteristic lesions of Typhoid fever.

Dr. Flint supposes that the miasm of contagion is much more abundant, or potent, at certain times and places, than when the disease ordinarily prevails. We know that this is the case with the miasm of small pox, for that seems to be almost governed in its virulence by determinate periods. "Or," says Dr. Flint, "it may be contended that the miasm, which generally is inoperative, becomes more efficient; not by any increase of its strength or quantity, but by the co-operation of other agencies, which favor its operation, or render individuals more susceptible to its influence."

We admit that the facts bear strongly towards the doctrine of contagion, but there are facts, even in this strong array, that seem to tell against it. We know of no contagion that pays respect to age; and a number of persons, of forty years and upwards, escaped an attack, though they were in the very focus of exposure. It strikes us that this is an obstacle to the fullness of the proof of contagion at North Boston. The facts and arguments of Professor Flint are stated with clearness, force and entire honesty.

We cannot quit this book without giving our cordial thanks to Professor Flint for his valuable contribution to the study of fever. He has acquitted himself with the highest degree of credit, and we commend his work as one of the best monographs that has appeared on the subject.

